# Cyclotide-rich fractions containing nanofibers by electrospinning: preparation, characterization and examination of antimicrobial activity

**DOI:** 10.55730/1300-0527.3468

**Published:** 2022-07-19

**Authors:** Elif Büşra BAŞ, Cemil KÜREKCİ, Orhan Murat KALFA, Muharrem AKCAN

**Affiliations:** 1 Department of Biochemistry, Faculty of Arts and Science, Kütahya Dumlupınar University, Kütahya, Turkey; 2 Department of Food Hygiene and Technology, Faculty of Veterinary Medicine, Hatay Mustafa Kemal University, Hatay, Turkey; 3 Department of Chemistry, Faculty of Arts and Science, Kütahya Dumlupınar University, Kütahya, Turkey

**Keywords:** Cyclotides, electrospinning, nanofibers, antimicrobial activity, drug delivery

## Abstract

In this study, antimicrobial nanofibers were produced with the mixtures of polyvinyl alcohol (PVA) and cyclotide-rich fractions by electrospinning method. After extraction, the first separation was carried out with C18 flash chromatography and then fractioned into five separate parts by reversed-phase high-pressure liquid chromatography (RP-HPLC). The molecular weights of cyclotides in each fraction were determined by quadrupole time-of-flight liquid chromatography-mass spectrometry (Q-TOF LC-MS). Cyclotide-rich fractions were mixed with 10% of PVA solution and nanofibers were produced from this biocomposite mixture by electrospinning method. The nanofibers were characterized by field emission scanning electron microscopy (FE-SEM), and it was observed that 100% peptide-containing nanofibers (cyclotide-rich fraction/10% PVA, w/v) had more regular fiber textures. The presence of the peptides in the nanofiber was also confirmed by analytical RP-HPLC, as the peptides in both peptide fractions and nanofiber solutions have the same retention times. The nanofibers produced with the fourth cyclotide-rich fraction showed activity against gram-positive bacteria (*Bacillus cereus*) in antimicrobial susceptibility test. As a result of these findings, cyclotide-containing nanofibers with antimicrobial activity can be produced for pharmaceutical research and development studies.

## 1. Introduction

Excessive and misuse of antibiotics have resulted in a major public health problem owing to the emergence and dissemination of antimicrobial resistance (AMR), which certainly necessitate alternative approaches for the treatment of infectious diseases [1,2]. Antimicrobial peptides (AMPs) are valuable molecules that can be utilized to overcome the AMR, since they have been shown to possess a broad range of antimicrobial activity [3–6]. AMPs can be obtained from animals, plants and various microorganisms such as bacteria and fungi [7]. They are mostly positively charged peptides with amphiphilic character and incorporate *α*-helices and *β*-sheets in their secondary structures [8].

**Figure 1:**
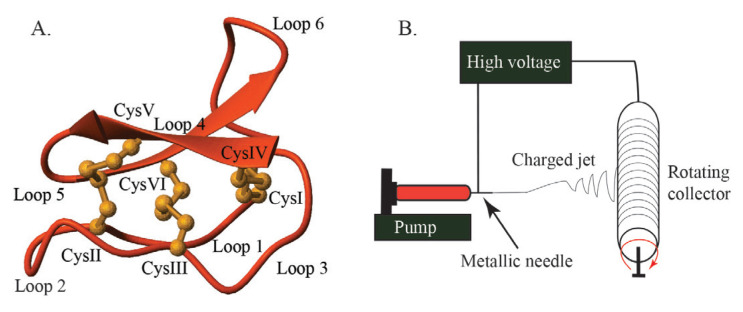
**A.** Three-dimensional structure of cyclotide kalata B1 (PDB code: 1NB1) with a cyclic cystine knot motif. The peptide backbone is shown in red, and the three disulfide bonds forming the cystine knot motif are shown in orange. Kalata B1 structure was prepared with MOLMOL [30]. **B.** Schematic illustration of an electrospinning device. It has a syringe with a metallic needle and a rotating collector. An automated pump is used to control the flow rate of the polymer solution and a high voltage is applied between the syringe needle and the collector to form the charged jet.

Cyclotides are a group of plant-derived disulfide-rich and highly stable cyclic peptides [9–11] with valuable bioactivities including insecticidal [12], anti-HIV [13], antimicrobial [14,15] and hemolytic [16] activities. They have been isolated from Violaceae, Rubiaceae, Cucurbitaceae, Solanaceae, Fabaceae and Poaecae plant families so far [17]. Cyclotides are about 30 amino acids long head-to-tail cyclic peptides with six conserved Cys residues that form three disulfide bridges [18]. The CysI-CysIV and CysII-CysV disulfide bridges form a ring shape, while the third one (CysIII-CysVI) passes through the middle of this ring and forms a cyclic cystine knot motif (CCK) (Figure 1A). Because of this motif, cyclotides are highly resistant to chemical, thermal and enzymatic effects compared to linear peptides [19]. The stable CCK motif and various bioactivities make cyclotides very promising candidates in drug design and development studies. Antimicrobial activities of cyclotides were first reported in 1999 for several synthetically produced cyclotides such as kalata B1, circulin A and B, and cyclopsychotride that showed antimicrobial activity against several bacterial strains [19]. In a recent study conducted in 2019, the antimicrobial activities of *Viola inconspicua* plant were investigated and antibacterial activity of some cyclotide fractions against gram-negative bacteria (*Escherichia coli* and *Klebsiella pneumoniae*) was reported [20]. Despite these valuable properties, antimicrobial peptides are sometimes need to be attached to a solid support such as metal surfaces, nanoparticles or nanofibers to overcome the obstacles in delivering of the antimicrobial activity to microorganisms and to reduce the cyctotoxic effects [21–23].

Electrospinning is an inexpensive and reliable material accumulation method to produce continuous nanosized fine fibers from polymer solutions (Figure 1B). Many different natural and synthetic polymers are used in the production of nanofibers. Natural polymers include collagen, cellulose, silk fibroin, keratin, gelatin, chitosan and alginate [24]. Polyethylene glycol (PEG), polycaprolactone (PCL), poly-(lactic-co-glycolic acid) (PLGA), poly(lactic acid) (PLA), polyglycolide (PGA), polyurethane (PU) and poly vinyl alcohol (PVA) can be given as examples for synthetic polymers [25]. PVA is a biocompatible and biodegradable polymer that can be dissolved in similar conditions as proteins (i.e. water soluble) and approved by the American Food and Drug Administration (FDA) for medical uses [26]. The molecular weight and concentration of PVA have important effects on nanofiber production and diameter [27]. Nanofibers are preferred due to their high porosity, large surface area, durability and ease of production for medical applications such as in drug release, tissue engineering, dental applications, wound dressings, and medical implants [28]. Moreover, it is also possible to control the bioavailability, encapsulation and drug release efficiency for a drug with nanofibers [28]. Electrospinning has been accepted as an innovative method in nanobiotechnology as it has been used to utilize the biological activities of peptides more efficiently. It provides a controlled release of peptides and helps to improve their activity. It can be also effective to reduce the toxicity and increase pharmaceutical properties such as bioavailability [29]. Additionally, when the peptide-containing nanofibers are examined, it has been reported that the peptides have provided stabilization, protection, elasticity and nanometer sizes to the fiber [25].

Drug delivery systems with antimicrobial properties have started to be important in wound dressings and band-aids [31–33]. In this work, new treatment agents were developed by taking advantage of the antimicrobial properties of cyclotides and nanofibers. Bioactive cyclotide-rich fractions were extracted from *Viola biflora* (yellow violet) flowers, which is one of the members of the Violaceae plant family, separated into five separate fractions by RP-HPLC and characterized by mass spectrometry. Then, cyclotide and PVA containing biocomposite nanofibers were produced with electrospinning method, characterized with FE-SEM and antimicrobial activities of the nanofibers were investigated by antimicrobial susceptibility test.

## 2. Material and methods

### 2.1. Cyclotide extraction, fractionation and LC-MS characterization

The flower part of the plant (*V. biflora*) was collected in Kütahya Province, in June 2018. Plant material was air-dried under shadow at 20 ± 2 °C and grounded to powder. A total of 60 g of flower powder was stirred in a 2.0 L of acetonitrile (ACN):H_2_O:formic acid (FA) (25:24:1, v/v/v) solution in a glass bottle for one day with a magnetic stirrer at room temperature [34]. After filtering the mixture through a glass wool and then with a filter paper, the filtrate was lyophilized using a freeze dryer (Telstar LyoQuest) to remove the solvents. The dried crude extract (approximately 34.13 g) was first purified with C18 flash chromatography. After conditioning of the column with 20 mL of Solvent A [20% ACN:0.05% trifluoroacetic acid (TFA) (v/v)], approximately 500 mg of crude plant extract dissolved in Solvent A was loaded to the column. Hydrophilic portions were eluted with 50 mL of Solvent A. The cyclotide-rich fraction was then eluted with 100 mL of Solvent B [80% ACN with 0.05% TFA (v/v)] and lyophilized. This step was repeated 66 times, resulting in 1.60 g of dry cyclotide-rich fraction. After first purification with C18 flash chromatography, cyclotide-rich part was re-fractionated by RP-HPLC (LC20, Shimadzu) on a C18 semipreparative column (Inertsil 20 × 250 mm, WP300, C18, 5 μm). For this fractionation, a 1% Solvent D gradient method (80 min) with a flow rate of 8 mL/min was used [Solvent C: H_2_O: 0.05% TFA (v/v), Solvent D: 90% ACN:10% H_2_O:0.045% TFA (v/v/v)]. HPLC chromatograms were recorded with a UV detector at 215 and 280 nm, and the fractions were collected into different containers for every 10 min during the 60 min (i.e. first fraction between 10 and 20 min, second fraction between 20 and 30 min, third fraction between 30 and 40 min, fourth fraction between 40 and 50 min, and fifth fraction between 50 and 60 min). Only Fractions 2, 3, and 4 were used as there is enough peptide material for further studies (>5 mg).

The cyclotide content of the collected fractions were then characterized by liquid chromatography-mass spectrometry (LC-MS). The LC-MS analyses of the samples were performed with a quadrupole time-of-flight liquid chromatography-mass spectrometer (Agilent G6530B Q-TOF LC-MS) equipped with a dual AJS ESI source. Nitrogen was used as both drying gas with 13 L/min flow rate and as nebulizer with 35 psi pressure. The nebulizing gas temperature was set at 325 °C, capillary potential 4000 V and the sheath gas flow rate was 12 L/min. Fragmentor voltage was set to 165 V and the skimmer voltage was 65 V of the MS TOF analyzer. One-milligram/milliliter sample solutions of Fractions 2, 3, and 4 were prepared in Solvent E and 50 μL of each solution was injected and the peptides were separated on an Agilent Poroshell 120 SB-C18 column (4.6 × 150 mm, 2.7 μm particle size). A 2% Solvent F gradient method with a flow rate of 0.3 mL/min for 60 min was used [Solvent E: H_2_O:0.1% FA (v/v) and Solvent F: 90% ACN:10% H_2_O:0.5% FA (v/v/v)]. The column temperature was adjusted to 25 °C. The mass spectra were recorded in positive ion mode and the mass/charge ratio was set to 500–3000 Da. LC-MS chromatograms and spectra were analyzed by Agilent Qualitative Analysis B.06.00 software.

### 2.2. Preparation of cyclotide containing nanofibers

#### 2.2.1. Preparation of PVA solution and production of PVA nanofibers

To determine the appropriate PVA percentage, polymer solutions having 5, 10, and 15 wt % of PVA in double distilled H_2_O were electrospun using an electrospinning system (Nano Web Elektrospin 350, Turkey) equipped with a 5 mL of 0.8 × 38 mm 21G sterile syringe and a fiber collector rotary plate (rotating at 3000 rpm/min and covered with aluminum foil). PVA was dissolved in distilled water and stirred in an ultrasonic bath at 80 °C for 2 h to ensure a complete dissolution. After PVA was completely dissolved in water, it was left to stir with a magnetic stirrer at room temperature over 24 h. The distance between the syringe needle tip and the collector was adjusted to 10 cm and the fibers were produced at 3.5 mL/h flow rate with 24 kV applied voltage. Based on the FE-SEM images, the optimum polymer concentration was determined as 10% PVA in H_2_O (w/v) as it has the better fiber texture and diameter compared to other nanofibers produced with different parameters.

#### 2.2.2. Production of cyclotide containing nanofibers

Firstly, 6.25 mg, 12.5 mg, 25.0 mg, and 37.5 mg of cyclotide mixtures from the second, third, and fourth fractions were mixed separately with 10% PVA solution to prepare 25, 50, 100, and 150 wt % of cyclotide-rich fraction/PVA mixtures, respectively. The solutions were stirred with a magnetic stirrer for 4 h at 20 ± 2 °C. Twelve cyclotide-rich fraction/PVA nanofibers in total were produced using the same parameters that was used to produce PVA nanofibers. The produced nanofibers are named as NFX-Y, where X is the fraction number and Y is the percentage of cyclotide-rich fraction in 10% PVA solution (w/v) **(**Table**)**.

#### 2.2.3. Characterization of nanofibers

Morphological characterization of nanofibers was performed with FEI Nova NanoSEM 650 Field Emission Scanning Electron Microscope (FE-SEM). Before the images were obtained by electron microscopy, samples were covered with gold and examined with FE-SEM at a voltage of 10 kV.

The presence of peptides in nanofibers were confirmed by comparing the retention times of peptides in nanofiber samples and peptide fractions alone by analytical RP-HPLC. For this purpose, 1 cm^2^ sample was cut from 100 wt % of cyclotide-rich fraction/PVA nanofiber mats (NF2-100, NF3-100, NF4-100) and from the nanofiber prepared only with PVA. One-milligram/milliliter solutions were also prepared from the second, third, and fourth RP-HPLC fractions. For the analysis, a method with a flow rate of 0.3 mL/min and a gradient of 2% Solution D was used for 35 min. The retention times of peptides and PVA of the nanofibers were compared to the retention times of peptides of the three RP-HPLC fractions.

### 2.3. Antimicrobial activities of nanofibers

Antimicrobial activities of the nanofiber produced only with 10% PVA and the peptide containing nanofibers (NF2, NF3 and NF4) were tested against a panel of gram-negative and gram-positive microorganisms with antimicrobial susceptibility test. Bacterial cultures were *Staphylococcus aureus* (ATCC 25923), *Enterococcus faecium (RSKK 623)*, *Enterococcus casseliflavus* (ATCC 700327), *Salmonella* Typhimurium (ATCC 14028), *E. coli* (ATCC 25922) and *Bacillus cereus* (ATCC 13061). Bacterial suspensions (10^8^ cfu/mL) were prepared from pure cultures grown on blood agar medium according to McFarland turbidity 0.5. From the prepared bacterial suspensions, inoculum was spread on the Müller Hinton agar plates. Nanofibers were cut in square pieces (approximately 1 cm^2^ in diameter) and then placed on the agars with inoculum. The prepared agar plates were incubated at 37 °C for 24 h. After incubation, the inhibition zones formed around the nanofiber samples were measured with a ruler. Standard antibiotics (ciprofloxacin; 5 mg/disc and ampicillin; 10 mg/disc) were also included in the assay as the control agents along with nanofiber samples.

## 3. Results

**Figure 2:**
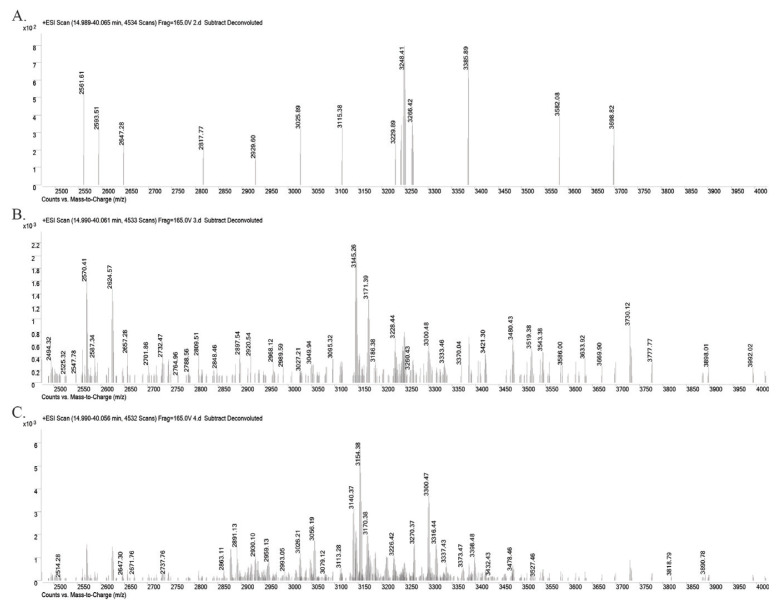
RP-HPLC chromatogram of the lyophilized cyclotide-rich part after C18 flash chromatography. Five different fractions were collected into different containers every 10 min. After lyophilization of the fractions, only Fractions 2, 3, and 4 were used for further studies as there was enough peptide sample for nanofiber production.

In Figure 2, a representative RP-HPLC chromatogram of the lyophilized cyclotide-rich part was shown. Only Fractions 2, 3, and 4 were used for nanofiber production as the hydrophobic cyclotides eluted between 20 and 50 min of the 80 min gradient method.

**Figure 3:**
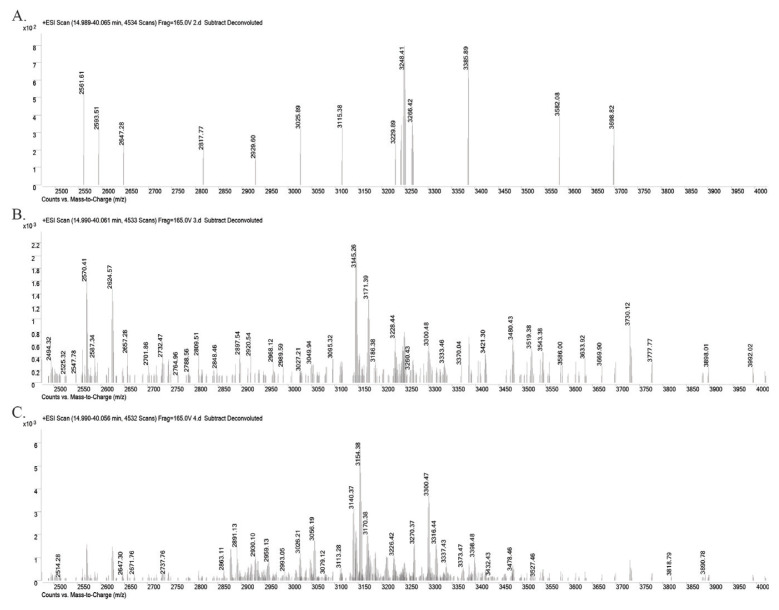
Deconvoluted mass spectra of **A.** Fraction 2, **B.** Fraction 3, and **C.** Fraction 4 after RP-HPLC. LC-MS chromatograms and spectra were analyzed by Agilent Qualitative Analysis B.06.00 software. Deconvoluted mass spectra are shown in the mass range of 2500–4000 Da since cyclotides fall within this mass range as in the previous literature [11].

The dried amount of the second fraction is 0.91 g, third fraction is 0.13 g, and fourth fraction is 0.19 g. Fractions 1 and 5 were not used as there was not enough peptides for further studies (<5 mg). After RP-HPLC, the molecular weights of cyclotides in Fractions 2, 3, and 4 were characterized by Q-TOF LC-MS. In Figure 3, the deconvoluted mass spectra of each three fractions were given. Molecular weights between 2500 and 4000 Da show that the fractions are rich in cyclotide content. For example, in Fraction 2, at least 11 cyclotide masses, in Fraction 3, 16 cyclotide masses, and in Fraction 4, 11 cyclotide masses were obtained.

**Figure 4:**
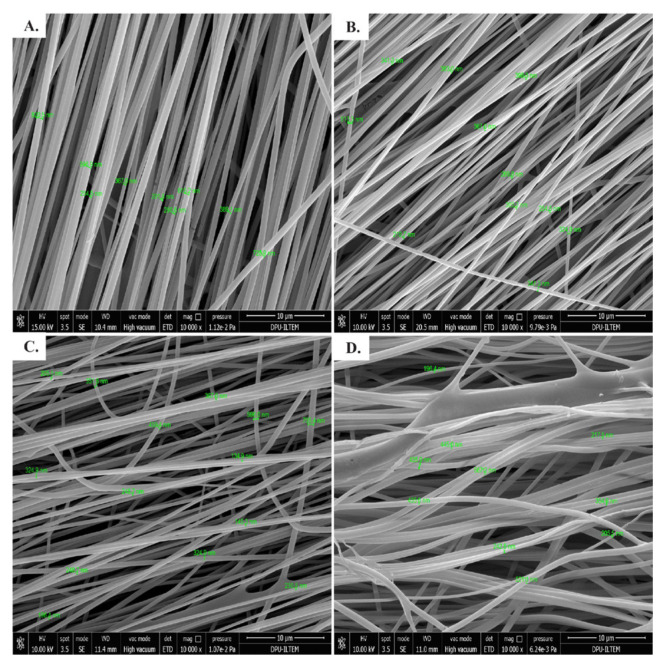
FE-SEM images of 100% w/v nanofibers (25 mg of cyclotide-rich fraction + 25 mL of 10% of PVA solution) of Fractions 2, 3, 4 and the nanofibers produced only with 10% PVA. **A.** FE-SEM image of nanofiber NF2-100 (Mag = 10000, fiber diameter range: 254.5–906.9 nm). **B.** FE-SEM image of NF3-100 (Mag = 10000, fiber diameter range: 210.7–513.3 nm). **C.** FE-SEM image of NF4-100 (Mag = 10000, fiber diameter range: 134.9–755.4 nm). **D.** FE-SEM image of PVA nanofiber (Mag = 10000, fiber diameter range: 196.4–567.2 nm).

Morphological characterization of nanofibers was performed with FE-SEM. In Figure 4, FE-SEM images of 100% w/v nanofibers of Fractions 2, 3, 4 and 10% PVA were shown as example because these nanofibers (NF2-100, NF3-100, NF4-100) have more regular fiber textures compared to others. The fiber diameter ranges between 134.9 nm and 906.9 nm for all nanofibers produced.

**Figure 5:**
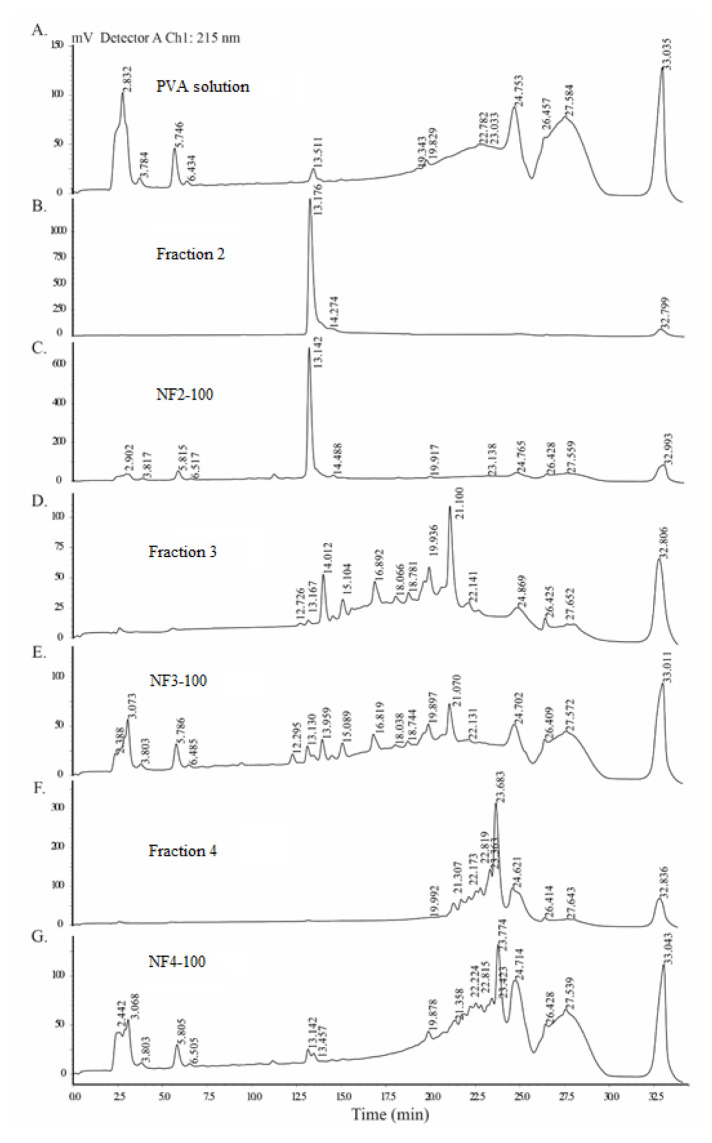
Analytical RP-HPLC chromatograms of the PVA solution, peptide fractions and the nanofibers (NF2-100, NF3-100, and NF4-100). **A.** Analytical RP-HPLC chromatogram of the nanofiber of 10% PVA solution alone. **B.** Analytical RP-HPLC chromatogram of Fraction 2. **C.** Analytical RP-HPLC chromatogram of NF2-100. **D.** Analytical RP-HPLC chromatogram of Fraction 3. **E.** Analytical RP-HPLC chromatogram of the NF3-100. **F.** Analytical RP-HPLC chromatogram of Fraction 4. **G.** Analytical RP-HPLC chromatogram of NF4-100. The similar retention times of the peptides in both the fractions and the nanofibers show that the nanofibers contain cyclotides.

In Figure 5, analytical RP-HPLC chromatograms are shown. PVA eluted at 23–30 min and cyclotides in Fraction 2 eluted at 12–15 min. For Fractions 3 and 4, cyclotides eluted at 12–23 and 19–25 min, respectively. Similar peptide retention times were also observed for the nanofibers NF2-100, NF3-100 and NF4-100 that contains Fractions 2, 3, and 4, respectively. Because the peptides have similar retention times, the production of the cyclotide containing nanofibers was achieved.

**Figure 6:**
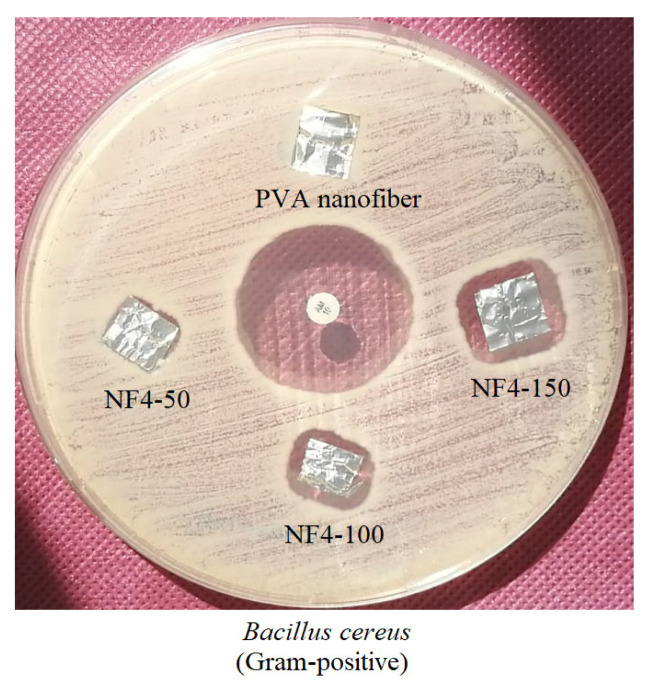
Antimicrobial activities of nanofibers NF4-50, NF4-100, and NF4-150 produced from Fraction 4/10% PVA solution and nanofiber produced only with 10% PVA solution. Cyclotide containing nanofibers did not show antimicrobial activity on *S.* Typhimurium (gram-negative) bacteria, but they showed activity on *B. cereus* (gram-positive) bacteria. NF4-150 nanofiber showed the highest activity compared to nanofibers NF4-100 and NF4-50 as it has the highest cyclotide content.

Antimicrobial activities of nanofibers on *S.* Typhimurium, *E. coli*, *S. aureus*, *E. faecium, E. casseliflavus* and *B. cereus* cultures were examined with antimicrobial susceptibility test. The nanofiber produced only with 10% PVA solution did not show any antimicrobial activity against all bacterial cultures tested. The results showed that the cyclotide-containing nanofibers also did not show any antimicrobial activity except the nanofibers produced with Fraction 4. While there was no activity observed for the nanofiber mats of Fractions 2 and 3 against *B. cereus*, the nanofibers produced with Fraction 4/10% PVA (NF4-50, NF4-100 and NF4-150) showed antimicrobial activity towards *B. cereus*. Furthermore, as in Figure 6, bacterial inhibition zone of NF4-150 is larger than the inhibition zones of both NF4-100 and NF4-50 and inhibition zone of NF4-100 is larger than NF4-50. This result shows that when the amount of peptide in the nanofiber mat increases, the diameter of the bacterial inhibition zone increases as well.

## 4. Discussions

In this study, we have used cyclic peptides (i.e. cyclotides) and PVA to produce nanofibers with antimicrobial activities. Cyclotides were extracted from *V. biflora*, one of the members of the largest plant sources of cyclotides, Violaceae [35]. In order to extract these cystine knot peptides, the second method reported by Mahatmanto et al. in 2014 was used [34]. In that report, five methods were studied. These methods include dichloromethane/methanol (1:1, v/v), ACN/H_2_O/FA (25:24:1, v/v/v), 20 mM sodium acetate (pH 5.0), 5 mM ammonium bicarbonate (pH 8.0) and the boiling water, respectively. The second extraction solution containing ACN/H_2_O/FA (25:24:1, v/v/v) resulted in higher crude extract yield and peptide content compared to other four methods. In Cybase (http://cybase.org.au/), a database that provides information about cyclic peptides (including cyclotides), there are nine wild type cyclotides from *V. biflora* have been reported [36]. In this study, many known and novel cyclotides that are in the mass range of 2500–4000 Da have been observed in the LC-MS analysis as reported in the literature. Each fraction has different number of cyclotides. The molecular weights of the peptides obtained in this study were compared with the data in Cybase, and some of the cyclotide masses (monoisotopic mass) obtained from the *V. biflora* were found to match these data. Also, different cyclotide masses not found in Cybase were also observed for the first time in this study. According to the RP-HPLC and mass spectrometry results, the *V. biflora* plant is a rich source of cyclotides. As reported in previous studies, the number of cyclotides obtained in each fraction is different from the literature because of the possible differences of expression levels or identities of cyclotides in plants can be vary depending on the season that the plant harvested and the plant parts used for the extraction studies [37,38].

After fractionation and characterization of cyclotide-rich fractions, nanofibers were produced by electrospinning. The distance between the needle tip and the collector plate was set to 15 cm at first. However, since the fiber formed in the collecting plate did not have a proper morphology and accumulation, the distance was updated as 10 cm. It has been observed that the fiber reaches and accumulates much better at this distance which is consistent with the results reported previously in which after determination of the appropriate parameters for electrospinning, 10 cm collector distance was used for the production of nanofibers of cathelicidin peptide LL37 with polyethylene oxide (PEO) and Magainin II peptide with poly(lactide-co-glycolide) (PLGA) or PLGA/gelatin mixtures [39, 40]. Magainin II peptide was immobilized on poly(lactide-co-glycolide) (PLGA) or PLGA/gelatin nanofiber membranes to examine the attachment and survival of bacteria on the membranes. Another example for a covalent immobilization of an antimicrobial peptide on a nanofiber is the immobilization of Cys-KR12 peptide, which is a shortened version of LL37 peptide, on the silk fibroin (SF) nanofibers by covalent bonds after a thiol-maleimide click chemistry [21]. The produced electrospun nanofiber membrane exhibited antimicrobial activity, facilitated the proliferation and differentiation of keratinocytes.

Unlike these last two studies, instead of covalent immobilization, we mixed cyclotide fractions with PVA first as in the production of antimicrobial nanofibers of pegylated LL-37 peptide (PEG-LL-37) with polyethylene oxide. Different concentrations of PEG-LL-37 peptide were used to examine the elimination of bacterial colonies at different rates. In our study, appropriate amount of peptide fractions was mixed with 10 wt % of PVA solution to prepare 25, 50, 100, 150, and 200 wt % of cyclotide-rich fraction/PVA mixtures. However, 200% w/v cyclotide-rich fraction/PVA mixture could not be prepared as this amount of peptide did not mix homogeneously with PVA solution which could be the result of the hydrophobic nature of cyclotides. In addition to the needle distance and the amount of active compound mixed with the polymer, the nanofiber diameters are also consistent with the literature values. The nanofiber diameters were reported 100–500 nm for PEG-LL-37 peptide-PEO nanofibers, 350 nm for Cys-KR12 peptide SF nanofibers, 715 nm for Magainin II peptide PLGA nanofibers, 356 nm tigecycline-loaded sericin/PVA composite fibers [21,31,39,40]. The maximum nanofiber diameters for NF2-100 are 906.9 nm, 513.3 nm for NF3-100 and 755.4 nm for NF4-100 were obtained in this study.

In previous studies, the antimicrobial activities of *V. odorata* Linn. and *V. tricolor* herb extracts obtained in solvents with different polarity were tested on different microorganism including especially *P. aeruginosa*, *S. aureus* and *C. albicans* [41–43]. In one of these studies where a cork borer with 6 mm diameter was used to prepare the wells and dimethyl sulfoxide (DMSO) was used as negative control, Gautam and Kumar reported that the inhibition zone diameter of *V. odorata* methanol extracts on *S. aureus* was 16 mm and 13 mm for *P. aeruginosa* [42]. In another study, Witkowska-Banaszczak et al. reported both ethanol and methanol extracts of *V. tricolor* have 0.15 mg/mL and 1.25 mg/mL minimum inhibitory concentration (MIC) values on both *S. aureus* and *B. cereus*, respectively [44]. For the gram (−) bacteria *E. coli* and *P. aeruginosa*, both ethanol and methanol extracts have 2.50 mg/mL and 1.25 mg/mL MIC values, respectively. These results show that the Violaceae plant extracts have antimicrobial activities on microorganisms that we have used in this study as well. However, instead of using the whole extracts for antimicrobial studies, we first separated the cyclotide-rich fractions and fractionated again into different parts. Then, three of these cyclotide-rich parts were used to prepare nanofibers with antimicrobial activities. The nanofibers produced with pegylated LL-37 peptide and PEO, the antimicrobial activities of these nanofibers were tested by disk diffusion method with *E. coli* and it is found that the antimicrobial activity of LL37 can be maintained on the nanofiber mat [40]. In this study, the nanofibers produced with Fraction 4/10% PVA (NF4-50, NF4-100 and NF4-150) showed antimicrobial activity towards *B. cereus*. Based on the previous studies, cyclotides with antibacterial activity can be listed as cycloviolacin O2 (cyO2), circulin A and circulin B (cirA and cir B), kalata B1, cyclopsychotride and cyclotides HB10 and HB11 from *Hedyotis biflora* [15,19,44,45]. In these studies, the most promising cyclotide is cyO2 as it had bactericidal activity against *Klebsiella pneumoniae*, *Pseudomonas aeruginosa*, *S. enterica* serovar Typhimurium LT2 and *E. coli*. However, the cyO2 was inactive against *S. aureus* and *E. faecium* [15]. On contrary, Kirkpatrick et al. reported that they could not detect any activity against *K. pneumoniae*, which attributed to the high strain variability among the bacterial species. However, they also reported the antibacterial activity of cyO2 on *A. baumannii* for the first time [44].

In conclusion, Viola plant family is a rich source of cyclotides, and the valuable properties of these peptides can be harnessed by using nanofibers in addition to grafting studies where the cyclotide framework is used as a stable scaffold for drug design and development. The nanofibers showed activity against gram-positive bacteria (*B. cereus*) in antimicrobial susceptibility test. As a result of these findings, cyclotide-containing nanofibers with antimicrobial activity can be produced for pharmaceutical research and development studies. It is also possible that the antibacterial activity is increased as the amount of peptide in the nanofiber increase.

## Figures and Tables

**Table:** Electrospinning parameters that are used to produce the nanofibers.

		Fraction % of cyclotide-rich fraction mixed with 10% PVA solution			

Fraction number nanofiber names	Parameters	25% (6.25 mg)	50% (12.5 mg)	100% (25.0 mg)	150% (37.5 mg)

Fraction 2 NF2-25, NF2-50, NF2-100, NF2-150	Rotation speed (rpm)	3000	3000	3000	3000
	Flow rate (mL/h)	3.5	3.5	3.5	3.5
	Voltage (kV)	24	24	24	24
	Needle-to-collector distance (cm)	10	10	10	10
	Syringe volume (mL)	5	5	5	5
	Electrical current (μA)	15–21	15–21	15–21	13–20
	Moisture (RH)	60%	65%	64%	40%
	Temperature (°C)	25	24	24	15–25

Fraction 3 NF3-25, NF3-50, NF3-100, NF3-150	Rotation speed (rpm)	3000	3000	3000	3000
	Flow rate (mL/h)	3.5	3.5	3.5	3.5
	Voltage (kV)	24	24	24	24
	Needle-to-collector distance (cm)	10	10	10	10
	Syringe volume (mL)	5	5	5	5
	Electrical current (μA)	13–19	12–19	10–19	14–22
	Moisture (RH)	33%–40%	31%–40%	32%–43%	35%–43%
	Temperature (°C)	19–25	22–26	22–25	20–24

Fraction 4 NF4-25, NF4-50, NF4-100, NF4-150	Rotation speed (rpm)	3000	3000	3000	3000
	Flow rate (mL/h)	3.5	3.5	3.5	3.5
	Voltage (kV)	24	24	24	24
	Needle-to-collector distance (cm)	10	10	10	10
	Syringe volume (mL)	5	5	5	5
	Electrical current (μA)	12–19	13–19	12–20	10–14
	Moisture (RH)	31%–40%	34%–43%	42%–48%	35%–39%
	Temperature (°C)	21–25	21–26	22–27	19–26
